# LC–MS profiling, in vitro and in silico C-ABL kinase inhibitory approach to identify potential anticancer agents from *Dalbergia sissoo* leaves

**DOI:** 10.1038/s41598-023-49995-1

**Published:** 2024-01-02

**Authors:** Hem N. Naik, Dilip Kanjariya, Shahnaz Parveen, Iqrar Ahmed, Abha Meena, Harun Patel, Ramavatar Meena, Smita Jauhari

**Affiliations:** 1 Department of Chemistry, SV National Institute of Technology, Surat, Gujarat 395007 India; 2 Molecular Bioprospection Department, CSIR-Central Institute of Medicinal and Aromatic Plants, Lucknow, Uttar Pradesh 226015 India; 3 Department of Pharmaceutical Chemistry, Prof. Ravindra Nikam College of Pharmacy, Gondur, Dhule, Maharashtra 424002 India; 4 Division of Computer Aided Drug Design, Department of Pharmaceutical Chemistry, R.C. Patel Institute of Pharmaceutical Education and Research, Shirpur, Maharashtra 425405 India; 5 Natural Product and Green Chemistry Division, CSIR-Central Salt & Marine Chemicals Research Institute, G. B. Marg, Bhavnagar, Gujarat 364002 India

**Keywords:** Biomarkers, Biochemistry, Chemistry, Computational chemistry, Molecular dynamics, Mass spectrometry, Computational chemistry, Screening, Computational chemistry, Screening

## Abstract

Belonging to the *Fabaceae* family, *Dalbergia sissoo*, a versatile plant, has gained prominence for its potent medicinal attributes, especially antipyretic, anti-inflammatory, and cardioprotective properties, as well as the use of its leaf juice in cancer treatment. Despite these recognized applications by natives and tribals, comprehensive insight into its biological activities and chemical composition remains limited. This study aimed to explore the cytotoxic potential of sequentially extracted leaf extracts from *Dalbergia sissoo* using various solvents, aiming to unveil the array of phytochemicals through LC–MS profiling. Among the extracts evaluated, the extract employing methanol:water extracting media (HN-2) appeared with the most remarkable results in both phytochemical diversity and biological activity. Furthermore, in vitro results of HN-2's in vitro anticancer efficacy were confirmed through in silico molecular docking and molecular dynamics simulation. These analyses demonstrated its ability to inhibit C-ABL kinase within leukemia K562 cells, directing that Dalbergia sissoo leaves serve as a bioactive agent reservoir. Consequently, this suggests that the *Dalbergia sissoo* plant is a potential source of bioactive compounds that can be used as a precursor for developing new cancer inhibitors, mainly targeting leukemia.

## Introduction

The plant kingdom is a vital source of biologically active phytochemicals that might find use in the food, cosmetic, and pharmaceutical sectors. Most plant-derived chemicals exhibit various biological activities such as antimalarial, anticancer, anti-inflammatory, hypoglycemic, and anti-asthmatic agents and antimicrobials. They are also used as antioxidants in the food sector to extend their proprietary shelf life^1–3^.

Chronic disease can be termed an illness that may develop slowly or rapidly but lasts longer and might be incurable and life-threatening^1^. One of the most common chronic diseases is cancer, which affects millions of people each year across the globe. Cancer Facts & figures 2022 report by the American Cancer Society estimated that an estimated 1.9 million new cancer cases will be diagnosed and 609,360 cancer deaths in the United States alone (https://www.cancer.org)^4^. Across the globe, this number will be much higher.

Researchers are finding ways to overcome this chronic disease in various fields. One of the prominent ways is natural products. Plants have proved to be an important natural source of anticancer therapy for many years^5,6^. For example, Vinca alkaloids are a significant class of anticancer therapies. The two primary naturally occurring active compounds derived from the Catharanthus roseus are vincristine and vinblastine. Under research in phase 2 studies, they showed potential action against leukemia, breast cancer, lung cancer, and many other types of cancer^2,3^. Similar to this, Podophyllotoxin, a highly well-known anticancer drug, was first isolated from the roots of Podophyllum spices in the 1980s, and its structure was confirmed in the 1990s. Etoposide and Teniposide, two therapeutically significant semi-synthetic analogs produced due to these inventions, have great potential to treat lymphoma and testicular cancers^7^. Similar to this, Camptotheca acuminate spices were used as a source to make anticancer drugs such as Topotecan, Irinotecan, Exatecan, and LE-SN-38, which are used to treat Epithelial cancers and found active on other various types of cancer cell lines^8–10^. Combretastin A-4, an anticancer drug derived from *Combretum caffrum*, is undergoing Phase-II clinical trials and is effective against various cancer cell lines^11^. A well-known component termed curcumin, originally derived from the plant Curcuma longa, is effective against colorectal and pancreatic cancer^12^. Schischkinnin, an anticancer from the plant Centaurea Schischkinnin, have been identified and proven to be very active on Colon cancer cell lines in vitro analysis^13^. Similarly, several different plants' polyphenols have shown impressive anticancer effect against several types of skin cancers^14^. Several flavonoids from the plant *Dryopteris erythrosora* were identified and showed in vitro anticancer effects^15^. Leaf extracts of *Marchantia convolute* exhibited its property against human liver and lung cancer cells^16^. Many more phytochemicals were identified and showed their remarkable properties against various cancer cell lines. Many more plants are still yet to be explored to identify new phytochemicals for various chronic diseases like cancer.

Plants of the genus *Dalbergia* have long been reported phytochemicals have been identified and shown remarkable beneficial for the traditional treatment of anticancer activity since older times. Many more plants must be studied to identify novel phytochemicals for chronic illnesses like cancer. Osteoarthritis, gonorrhea, and rheumatic problems^17^. Phytochemical analysis of the *Dalbergia* genus showed many compounds, including flavonoids, benzophenones, styrene, and terpenoids^17,18^. From the literature survey, *Dalbergia sissoo* exhibited various biological activities from various parts of the plant, such as antimicrobial, neural, cardiac, antiparasitic, antidiabetic, anti-inflammatory, analgesic, Osteogenic, dermatological, gastrointestinal, and reproductive^19^.

*Dalbergia sissoo* may be found in natural and planted forests across India, Pakistan, Afghanistan, Bangladesh, Iran, Iraq, Thailand, Indonesia, Malaysia, Ethiopia, Nigeria, Sudan, Zimbabwe, Kenya, Tanzania, and the United States^20^. *Dalbergia sissoo* is broadly used in folk medicine for several diseases and other applications^21^. Heartwood extract in milk was given for fevers, while bark extract was used as an anti-inflammatory in piles sciatica and as a blood cleanser. Externally, the oil was used to treat skin disorders and infected ulcers. The wood served as an anthelmintic, antileprotic, and cooling agent. Arial portions were used to treat spasms, as an aphrodisiac, and as an expectorant. The extract of the leaves has been utilized as an antidiabetic, antioxidant, anticancer, analgesic, antipyretic, and treatment for jaundice. Flowers were used to treat skin disorders, as a blood purifier, and to boost immunity^22^.

Molecular docking and simulation studies were conducted against the C-ABL kinase protein to validate the anticancer activity. C-ABL kinase plays a vital role in the regulation of the actin cytoskeleton and the regulation of the cell cycle. They also act as a vital developer of the nervous system. For these reasons, targeting and inhibiting this particular enzyme ultimately gives us the anticancer effect of the particular cell^23,24^.

**Figure 1:**
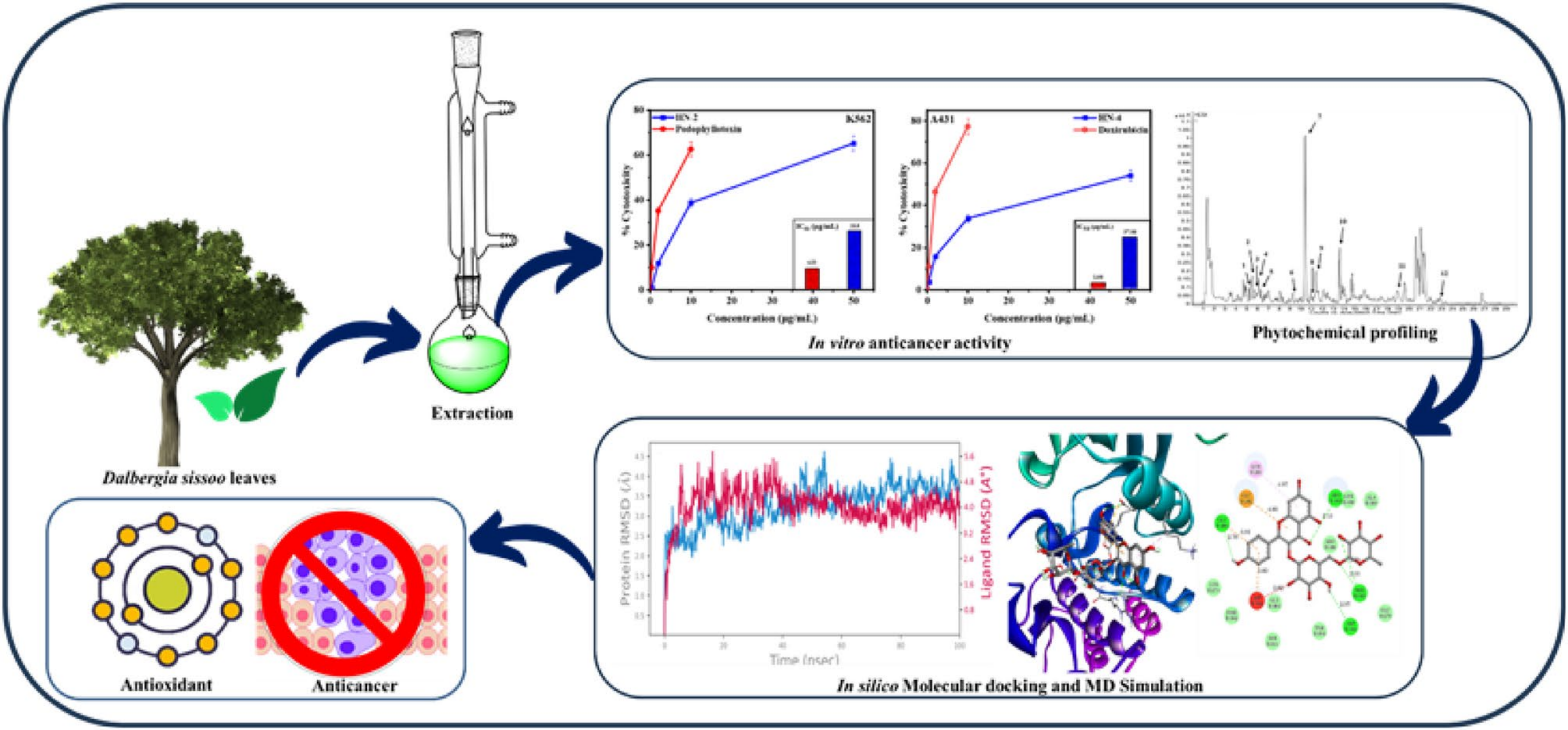
Graphical illustration of the current investigation.

Our lab is involved in identifying potent phytochemicals from natural sources and in biomass waste minimization^21,25^. As seen in Fig 1, this study aims to explore the utilization of *Dalbergia sissoo* leaves in traditional cancer treatment and to ascertain the potential of its phytochemical compounds through comprehensive extraction and screening from these leaves. We aim to investigate their potential for treating leukemia using both in vitro and in silico approaches, thereby seeking to identify novel anticancer drugs.

## Experimental

### Materials

Methanol HPLC grade, Sulfuric acid Emplura, Hydrochloric acid Emplura, and Isopropyl alcohol were purchased from Merck Millipore, Mumbai, India. 2-diphenylpicrylhydrazyl (DPPH reagent), and Ascorbic acid AR were purchased from Sigma Aldrich, Mumbai, India. α-naphthol AR, Lead acetate AR, bromine water AR, Potassium hydroxide AR, Ammonium hydroxide AR, and Sodium hydroxide AR were acquired from SRL, Mumbai, India. Absolute alcohol was acquired from Advent Chembio, Mumbai, India. Dragendorff's reagent, Hager's, and Wagner's reagent were purchased from Loba Chemie, Mumbai, India. All chemicals were used as received.

### Collection and preparation of extracts

The fresh leaves of the plant *Dalbergia sissoo* were collected after obtaining permission from the landowner who cultivated the plant, which is located in the village Vegam (20°52′06.8" N 73°01′07.7" E), in district Navsari, Gujarat, Western India. The additional permission to collect and work on the plant *Dalbergia sissoo was* taken from the Forest Officer of the Range Forest Office Chikhli, Navsari, Valsad North Forest Range. All the experimental procedures, including the collection and field studies, were conducted as per the standard guidelines as described in standard literature, "Dr. Bimal Desai, ASPEE College of Horticulture and Forestry, Navsari Agricultural University, Navsari, India authenticated a Handbook of Field and Herbarium Methods"^26^ and the material. The same specimen has been stored at the Herbarium (Voucher Specimen No: HNN/SVNIT/DS-1-3/2023). The collected material leaves were rinsed with distilled water to eliminate dust particles and shade-dried at room temperature (25 °C). Dried leaves were crushed using a mixer grinder and stored at room temperature until the extraction process was carried out.

Four different extracts were achieved by employing sequential extraction in different solvent systems (Table 1), and the extraction procedure was done as per standard methodology as described in the literature^26^. The material was first defatted using *n*-Hexane at room temperature (25 °C). A fixed amount of defatted powdered (50 g) was sequentially extracted using pure methanol as extracting solvent by Soxhlet extraction Method using a 1:10 material to a solvent ratio (HN-1)^25,27^, then by methanol:water mixed solution (HN-2) (ratio 9:3). Residual leaf mass was then treated with cold & hot water to extract water-soluble ingredients to check their bioactivity. The cold-water extract (HN-3) was used in the maceration Method ^28^ and the hot-water extract (HN-4) was collected using the Soxhlet method. After extraction, solvents were removed using a rotary evaporator for methanolic extracts, and water was removed using the freeze-drying method. All four derived solid extracts were stored in air-tight vials in a refrigerator.

**Table 1:** List of examined *Dalbergia sissoo* leaf extracts.

Name of extract	Extracting solvent
HN-1	Methanol
HN-2	Methanol:water
HN-3	Cold water
HN-4	Hot water

### Preliminary phytochemical screening

In line with the protocols of the study^29^, a small amount of the dry extract was utilized for phytochemical analyses for components such as carbohydrates, saponins, coumarins, steroids, glycosides, tannins, flavonoids, alkaloids, gum, and quinones. 1.0 g of each extract was dissolved in 10 mL of their extracting solvents and filtered (using Whatman No. 1 filter paper), and filtrate was used for the phytochemical tests.

### DPPH free radical scavenging capacity

Antioxidants can be water-soluble, lipid-soluble, insoluble, or attached to cell walls^30^. Methanol and ethanol are the most commonly used solvents for evaluating the radical scavenging activity of 1,1-diphenyl-2-picrylhydrazyl (DPPH)^31^. The DPPH was obtained from Sigma-Aldrich, gallic acid AR, and methanol HPLC was purchased from Finar Chemicals. DPPH radical scavenging activity was determined using the standard method with slight modifications^32^.

The DPPH scavenging potential was determined by 0.5 mL of plant extract solutions (HN-2, HN-3, and HN-4) in five different concentrations, i.e., 10, 20, 50, 70, and 100 µg/mL. Methanol (3 mL) and 0.5 mM DPPH solution (0.3 mL) were added to each, and the absorbance was measured after incubating the mixture for 45 min at 517 nm using a UV/VIS Spectrophotometer. An equal amount of DPPH and methanol were used as standard and black, respectively. Based on the percentage inhibition (calculated using Eq. 1), IC_50_ was calculated using the standard curve method.

1 $$Inhibition (\%)=\left[({A}_{Control}-{A}_{Sample})/{A}_{Control}\right]\times 100$$

where A_control_ is the absorbance of negative control at the moment of solution preparation and A_sample_ is the absorbance of a sample after 45 min.

### In vitro anticancer activity

The MTT experiment was performed twice using the K-562 cell line derived from leukemia^33^, PC3 cell lines representing human prostate cancer cells^34^, epidermoid (skin) cancer cell line A431 is used to perform the particular cancer inhibitory evaluation^35^. Cell lines NCIH-460 and A549 are widely used to investigate the anticancer potential of compounds against lung cancer^36,37^ and HEK-293T is a cancer cell line derived from human kidney malignant tumors^38^. which were acquired from the National Centre for Cell Sciences (NCCS), Pune, India. The cells were grown in a CO_2_ incubator at 37 °C, with 95% humidity in a high glucose DMEM supplemented with 1% Ab/Am and 10% FBS, pH 7.2–7.4. About 103-well plates were used for seeding, and the plate was incubated for 24 h. After incubation, cells were treated with varied doses of *Dalbergia sissoo* leaf extracts and incubated for another 24 h before being treated with 10 µl of MTT dye (5 mg/mL). The plate was then kept in the dark area for four hours at 37 °C, and absorbance was recorded in a spectrophotometer at 570 nm. The results were expressed regarding percent cytotoxicity, calculated concerning the control^39^.

2 $$\%Viability=\frac{\left[Sample(OD)-Zero day\,\, control (OD)\right]}{\left[Control(OD)-Zero day\,\, control(OD)\right]}\times 100$$

3 $$\% Cytotoxicity=\left(100-\%viability\right)$$

The IC_50_ values for the extracts were obtained by taking the concentration of the extract's percentage cytotoxicity by half of the initial cell population^40^.

### Phytochemical profiling

High-Resolution Liquid Chromatography–Mass Spectrometry (HRLC–MS) is a vital analytical tool for characterizing the phytochemical composition within plant extracts. In the context of our study, which focuses on four distinct crude extracts derived from the leaves of *Dalbergia sissoo*, it is noteworthy that extract HN-2 has exhibited the highest level of anticancer activity, as elucidated in Table 4 and Supplementary Table S1.

To get detailed information on the investigation of extract HN-2, we conducted HRLC-MS profiling. The primary objectives behind this profiling were: firstly, to get the comprehensive phytochemical profile of extract HN-2, and secondly, to facilitate the in silico studies. Additionally, we aimed to get an idea about the specific phytochemical constituents responsible for the observed anticancer properties. This strategic use of HRLC-MS analysis enables us to explore the molecular constituents and their potential mechanisms of action within extract HN-2, thereby enhancing our understanding of promising anticancer activities.

Analysis was performed for the extract having the highest anticancer potential (HN-2) using a Q-TOF Mass Spectrometer (Agilent Technologies) equipped with a column ZORBAX Eclipse Plus C18, Narrow Bore 2.1 × 150 mm, 5-micron; applying the following gradient at flow rate of 300 µL/min. The injection volume was 5.00 μL (Injection with Needle Wash). The thermostatic autosampler was kept at 40 °C^41^. 100% water was used in Channel A, and for Channel B, 100% Acetonitrile was used at various time intervals for a maximum of up to 30 min at modified constant flow and pressure of 300 µL/min and 1200.00 bar^42^.

### Molecular docking study

Molecular docking was performed for the screened bioactive phytochemicals by HRLC-MS analysis (Table 4 and Fig. 5) with a crystal structure of the C-ABL kinase domain of cancer cell line K562 (PDB ID: 1IEP) using auto-dock vina wizard with Podophyllotoxin as standard drug^43^. The docking procedure such as ligand optimization and preparation, protein preparation, visualization, etc.) can be found in previous studies because the same settings were used for the examined systems and the active site-specific docking, the grid (60 Å × 60 Å  × 60 Å) and grid center (x = 7.154, y = 96.256, and z = 59.452) were used^44^.

### Molecular dynamics (MD) simulation study

The MD simulations were conducted using the Schrödinger Desmond 6.9.137 MD simulation program (version 2022-1) on a Z4 HP workstation with the Linux operating system (Ubuntu 18.0.4 LTS). The workstation was equipped with 24 CPUs, 4 GPUs, and an Intel(R) Xeon(R) CPU E5-2690 v3 @ 2.60 GHz^45^.

The MD simulations were performed on the best docking pose of Phytochemical Biorobin in a complex with the C-ABL kinase Domain (PDB ID: 1IEP). The OPLS3e force field was employed, and the SPC solvation model was utilized for solvation effects. The simulation parameters and experimental details were followed exactly as described in previous studies^46,47^. The duration of the MD simulation was 100 ns, and trajectory snapshots were captured at 100 ps intervals for analysis. To predict the binding orientation and stability of the ligand using the MD trajectories, Desmond's Simulation Interaction Diagram (SID) was employed.

### Drug likeness and ADMET study

The screened phytochemicals' Drug Likeness and ADMET characteristics were determined using the PreADME/Tox server^25^.

## Results and discussion

### Preliminary phytochemical screening

The results of the HN-1, HN-2, HN-3, and HN-4 extracts phytochemical analysis of the leaves of *Dalbergia sissoo* are presented in Table 2. The leaf extracts of *Dalbergia sissoo* in different solvents showed the presence of various phytochemicals. This can be further connected with the tribal and retro uses of these leaves in South Gujarat, India, as they are potent cytotoxic, analgesic, antipyretic, and therapeutically very useful^48^.

**Table 2:** Preliminary phytochemical screening of *Dalbergia sissoo* leaf extracts.

Entry	Name of phytochemical	HN-1	HN-2	HN-3	HN-4
1	Alkaloid	+	+	−	+
2	Carbohydrate	+	+	−	+
3	Saponin	−	+	+	+
4	Flavonoid	+	+	+	+
5	Steroid	+	+	−	−
6	Tannin	+	+	−	−
7	Gum	−	−	−	−
8	Coumarin	+	+	−	−
9	Quinone	−	−	−	−
10	Proteins and amino acids	+	+	−	+
11	Terpenoid	−	−	−	−
12	Cardiac glycosides	+	+	−	−
13	Anthraquinones	+	−	−	−

( +) = present and (−) = absent.

### DPPH free radical scavenging capacity

Free radicals are linked to oxidative damage, while antioxidants are reducing agents that restrict oxidative harm to biological structures by offering electrons to free radicals and rendering them inactive^49^. When oxygen interacts with specific molecules, it generates free radicals. Once formed, the primary threat arises from the potential harm they can cause when interacting with vital cellular components such as DNA, proteins, and the cell membrane^50^. These free radicals engage with antioxidants, nullifying their destructive potential before any damage is initiated^51^. In plant biology, various secondary metabolites are synthesized, many acting as antioxidants^52^. A Current study was done to investigate the in vitro free-radical scavenging potential of *Dalbergia sissoo*.

**Figure 2:**
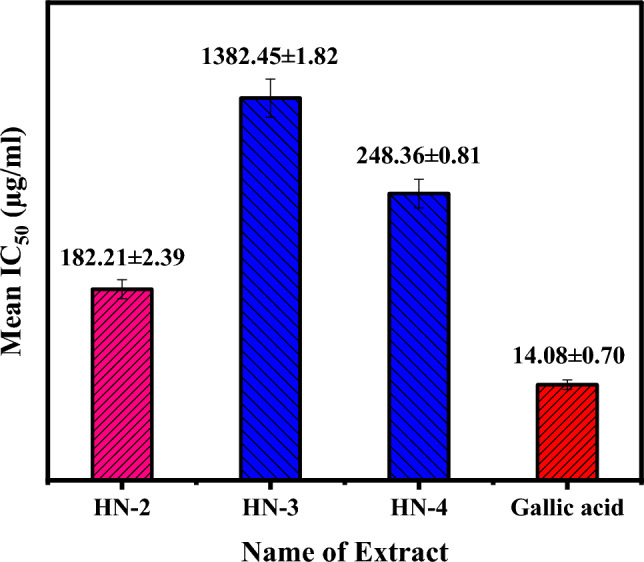
Comparison of the mean IC_50_ value for antioxidant activity of various extracts.

Rijhwani et al. have reported in vitro antioxidant activity of the plant Dalbergia sissoo's ethanolic and methanolic leaf extracts using the same technique with up to 300 ppm concentration of the extracts. The IC_50_ value for the ethanolic extract was determined to be 106.3, whereas the methanolic extract had a value of 815.53. The antioxidant potential was studied for three HN-2, HN-3, and HN-4 extracts only, and the percentage inhibition and means IC_50_ values of the three-leaf extracts of plant *Dalbergia sissoo* by DPPH scavenging analysis are depicted in Fig. 2 and Table S4. Among the three leaf extracts, the HN-2 demonstrated the highest percentage inhibition at a concentration of 100 ppm, i.e., 15.80 ± 0.79. This could be due to the phytochemicals present in HN-2 that are suitable for inducing higher inhibition. Under identical conditions at a 100 µg/mL concentration, the cytotoxicity percentage of the HN-3 was low (5.08 ± 0.79) compared to HN-2. This is relatively low and may produce fewer phytochemicals with cold water extraction. These outcomes (Tables 2, Tables S2 and S4) confirmed that the methanol:water solvent protocol is most suitable for the maximum phytochemical extraction from the *Dalbergia sissoo* leaves.

### In vitro anticancer activity

**Figure 3:**
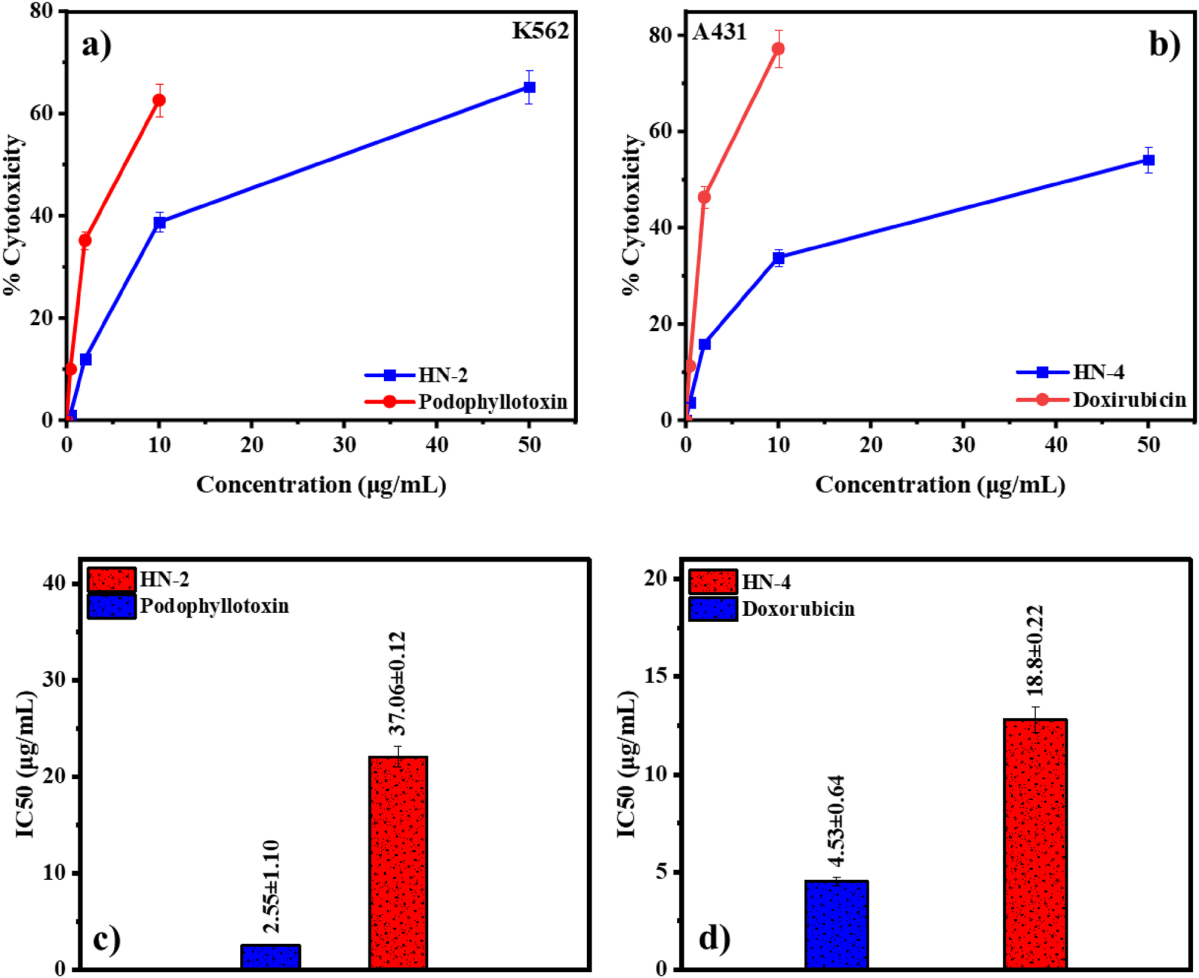
**(a)** Concentration versus percentage cytotoxicity of extract HN-2 against K562 cell line, (**b)** concentration versus percentage cytotoxicity of extract HN-4 against A431 cell line, (**c)** graphical representation of IC_50_ value of HN-2 in comparison to standard podophyllotoxin, and (**d**) graphical representation of IC_50_ value of HN-4 in comparison to standard doxorubicin.

Anticancer evaluation of leaf extracts and standard drugs against the cell as mentioned above lines performed as described in the experimental section. After taking absorbance with a UV–vis spectrometer, percentage viability was calculated using Eq. (2). After that, the percentage cytotoxicity of each extract was calculated at different concentrations by Eq. (3). Percentage cytotoxicity and IC_50_ of active extracts at different concentrations is represented in Table 3 and Fig. 3. Here, Podophyllotoxin was used as standard against the K562 cell line, and Doxorubicin was used against the remaining cell lines. The average cytotoxicity of remaining extracts on all cell lines is tabulated in Table S1. Based on the in vitro anticancer results, HN-2 was considered the most active against the K562 cell line and screened further by LC–MS to get the Phyto profile.

**Table 3:** Percentage cytotoxicity and IC_50_ values of active plant extracts on different cell lines.

Entry	Concentration (µg/mL)	Cytotoxicity (%)	IC_50_ (µg/mL)
K562 cell line			
HN-2	50	65.19 ± 1.19	**18.8 ± 0.22**
	10	38.76 ± 8.42	
	2	12.05 ± 2.61	
	0.4	1.12 ± 3.29	
Podophyllotoxin	50	–	4.53 ± 0.64
	10	62.62 ± 0.47	
	2	35.20 ± 0.96	
	0.4	9.98 ± 10.54	
A431 cell line			
HN-4	50	54.13 ± 0.31	37.06 ± 0.12
	10	33.84 ± 0.45	
	2	15.89 ± 0.32	
	0.4	3.72 ± 0.98	
Doxorubicin	50	–	2.55 ± 1.10
	10	77.23 ± 3.17	
	2	46.37 ± 6.19	
	0.4	11.29 ± 1.74	

Significant values are in bold.

### Phytochemical profiling

**Figure 4:**
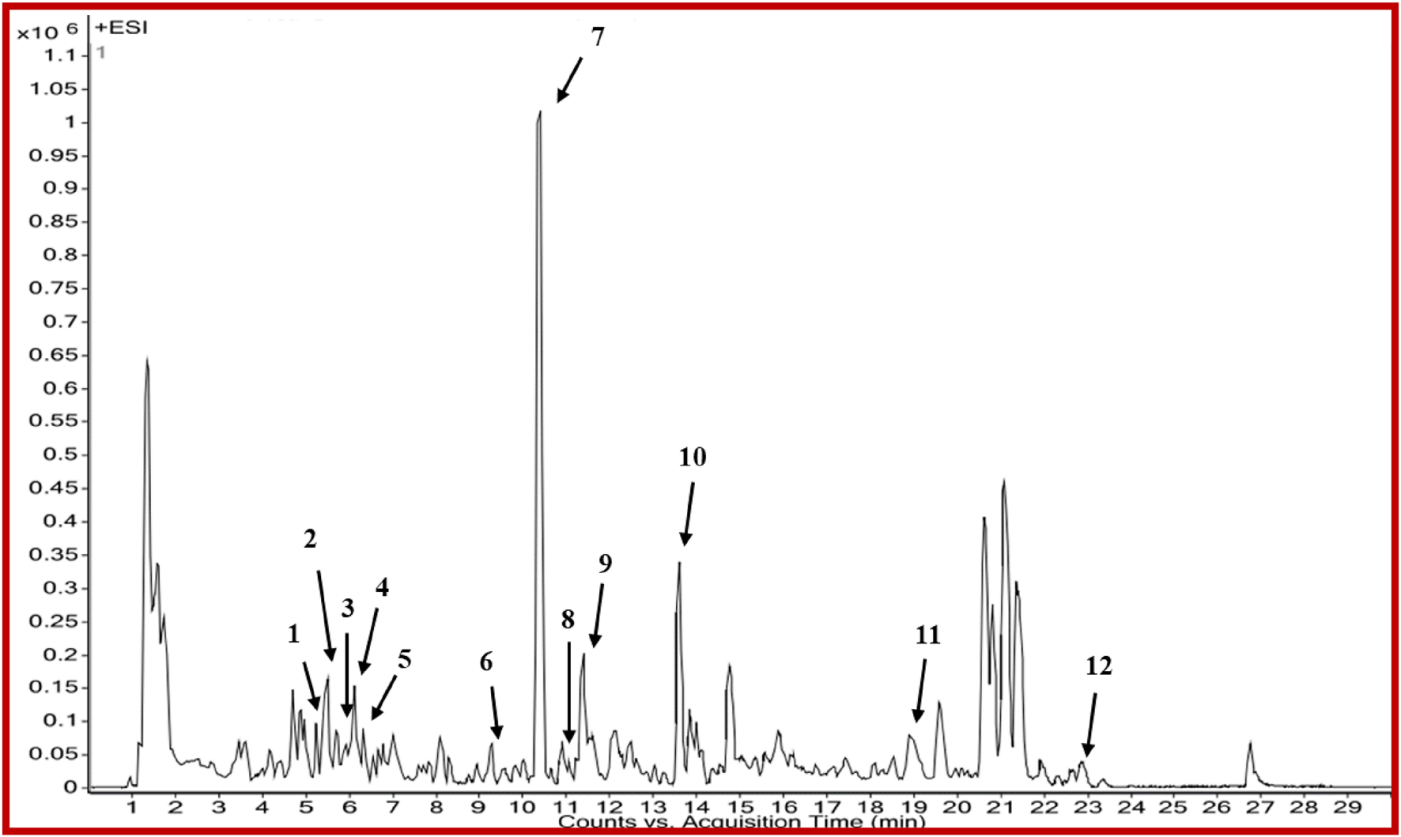
HRLC-MS spectrum of bioactive extract HN-2 (numbers belong to phytochemicals mentioned in Table 4).

Coupling such techniques and biological evaluation can give a broad idea regarding developing novel inhibitors for chronic diseases such as cancer. Based on the above-described analysis, the HRLC-MS analysis was performed to understand the plausible phytochemicals that exhibited good antioxidant and anticancer evaluation. Among all extracts, HN-2 showed excellent bioactivities against all tested cell lines. Hence, HN-2 was analyzed using HRLC-MS (Fig. 4) to identify phytochemicals responsible for high anticancer activity against K562. By comparing the fragmentation pattern in the positive mode (Fig. 4) against the spectra of the reference compounds and literature, a total of 12 bioactive compounds shown in Table 4 were tentatively identified.

**Table 4:** List of phytochemicals identified from LC–MS profiling with retention time and molecular weight.

Entry	Name of phytochemical	Retention time (min)	Molecular weight (g/mol)
1	Maculosin	5.12	260.11
2	Fraxetin	5.41	208.03
3	Isoorientin 7-glucoside	6.12	610.15
4	Ismine	6.12	257.10
5	Biorobin	6.18	594.16
6	Cirsimaritin	9.27	314.08
7	Manumycin A	10.34	550.26
8	Celereoin	11.01	262.08
9	Sayanedine	11.44	298.08
10	Armillarin	13.67	414.20
11	Irinotecan	19.12	586.27
12	Euphornin	22.93	584.30

All the tentatively identified phytochemicals were previously reported from various sources other than *Dalbergia sissoo* and have the potential to inhibit cancer cell growth or be used as anticancer agents (Table S2)^39,48,53–62^. The pharmacological activity of the extracts depends upon the presence of phytochemicals and the amount of those phytochemicals. In vitro*,* antibacterial, and antifungal evaluation results (As included in the supporting file) of these phytochemical extracts also show that HN-2 is the best extraction protocol for bioactive phytochemicals. Mass spectrums of identified phytochemicals are enclosed in supporting files from Fig S1 to Fig S12.

### Molecular docking study

**Figure 5:**
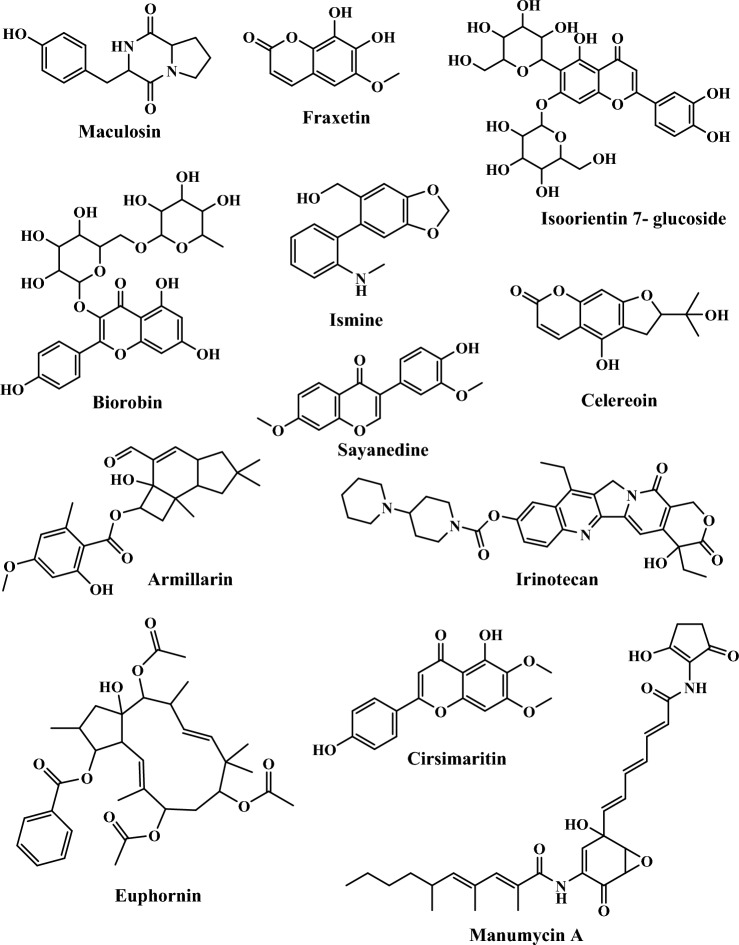
Chemical structures of phytochemicals identified by LC–MS profiling and used for molecular docking study.

In vitro*,* anticancer study findings indicated that HN-2 exhibits the highest inhibitory action against the K562 cell line, which was examined by the HRLC-MS study to identify the present bioactive phytochemicals. The molecular docking method evaluated the in silico binding interactions and the inhibitory potential of screened phytochemicals. This study acquired the crystal structure of the C-ABL kinase domain of cancer cell line K562 (PDB ID: 1IEP) from the PDB server (https://www.rcsb.org/). The screened phytochemicals have been identified from plants other than *Dalbergia sissoo* and have the reported cancer inhibitory potential listed in Table S2 (Fig. 5).

**Figure 6:**
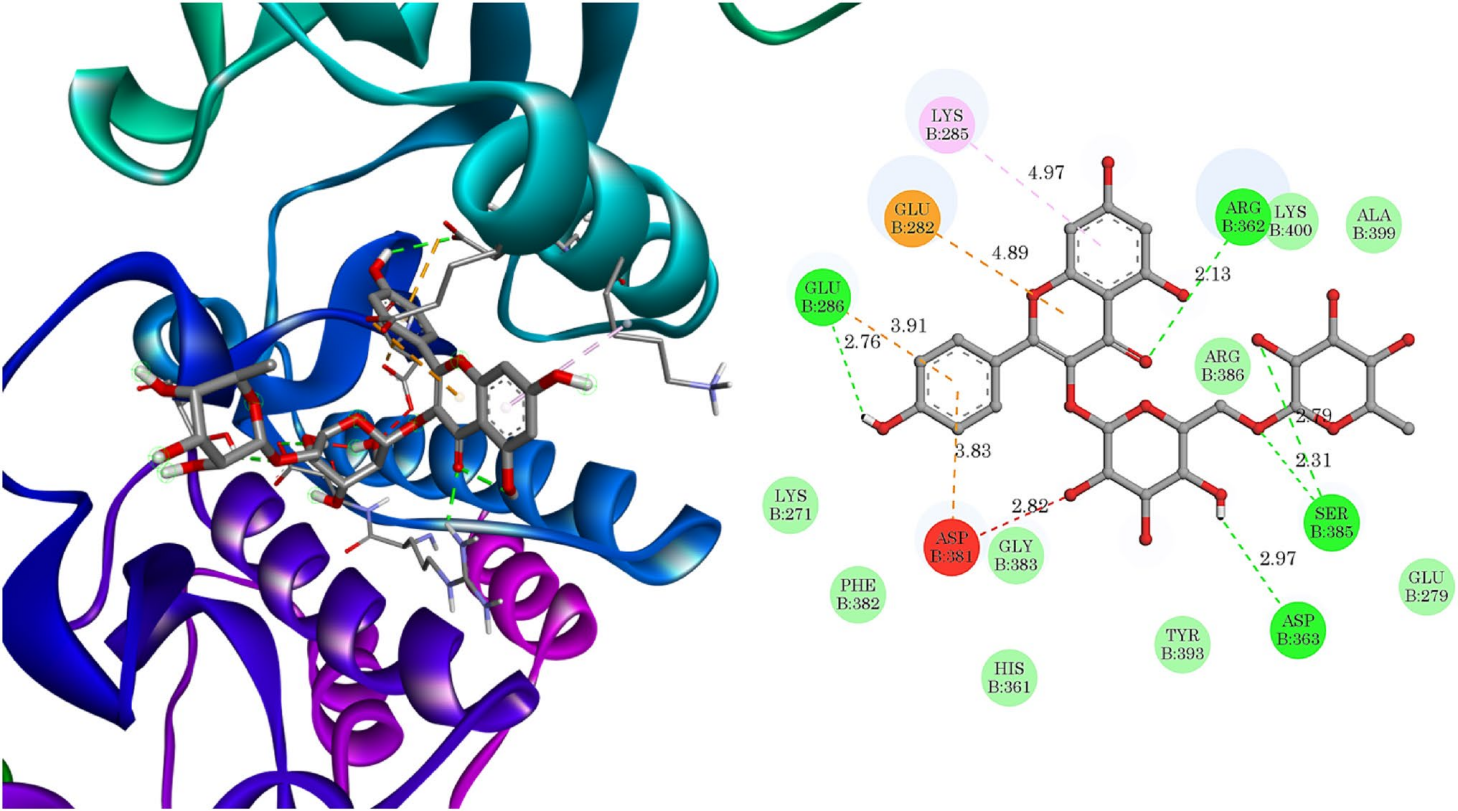
Binding interaction of biorobin in the active site of the C-ABL kinase domain of cancer cell line K562 (PDB ID: 1IEP).

All the bioactive phytochemicals docked nicely onto the active site of the K562 protein, with docking scores ranging from −9.0 to −6.0 kcal/mol, as enumerated in Table 5. Biorobin had the highest dock score of −9.0 kcal/mol among the tested bioactive compounds, as represented in Fig. 6. On the other hand, the standard inhibitor Podophyllotoxin had a docking score of −7.4 kcal/mol. Among all bioactive phytochemicals, Biorobin exhibited strong bonding interactions with the active site of kinase domain with strong hydrophilic bonds (2-Ser385 2.31 and 2.79 Å, Asp363 2.97 Å, Asp381 2.82 Å, 2-Glu286 2.76 and 3.91 Å, and Arg362 2.13 Å), two hydrophobic bonds (Glu282 4.89 Å and Lys285 4.97 Å).

**Table 5:** Docking score of Identified phytochemicals in the C-ABL kinase inhibitor (PDB ID: 1IEP).

Name of phytochemical	Docking score (kcal/mol)
Biorobin	**−9.0**
Irinotecan	**−**8.6
Isoorientin 7-glucoside	**−**8.4
Armillarin	**−**7.9
Euphornin	**−**7.4
Maculosin	**−**7.4
Celereoin	**−**7.3
Cirsimaritin	**−**7.0
Manumycin A	**−**7.0
Sayanedine	**−**7.0
Ismine	**−**6.6
Fraxetin	**−**6.0
Podophyllotoxin	**−**7.4

Significant values are in bold.

### Molecular dynamics (MD) simulation study

**Figure 7:**
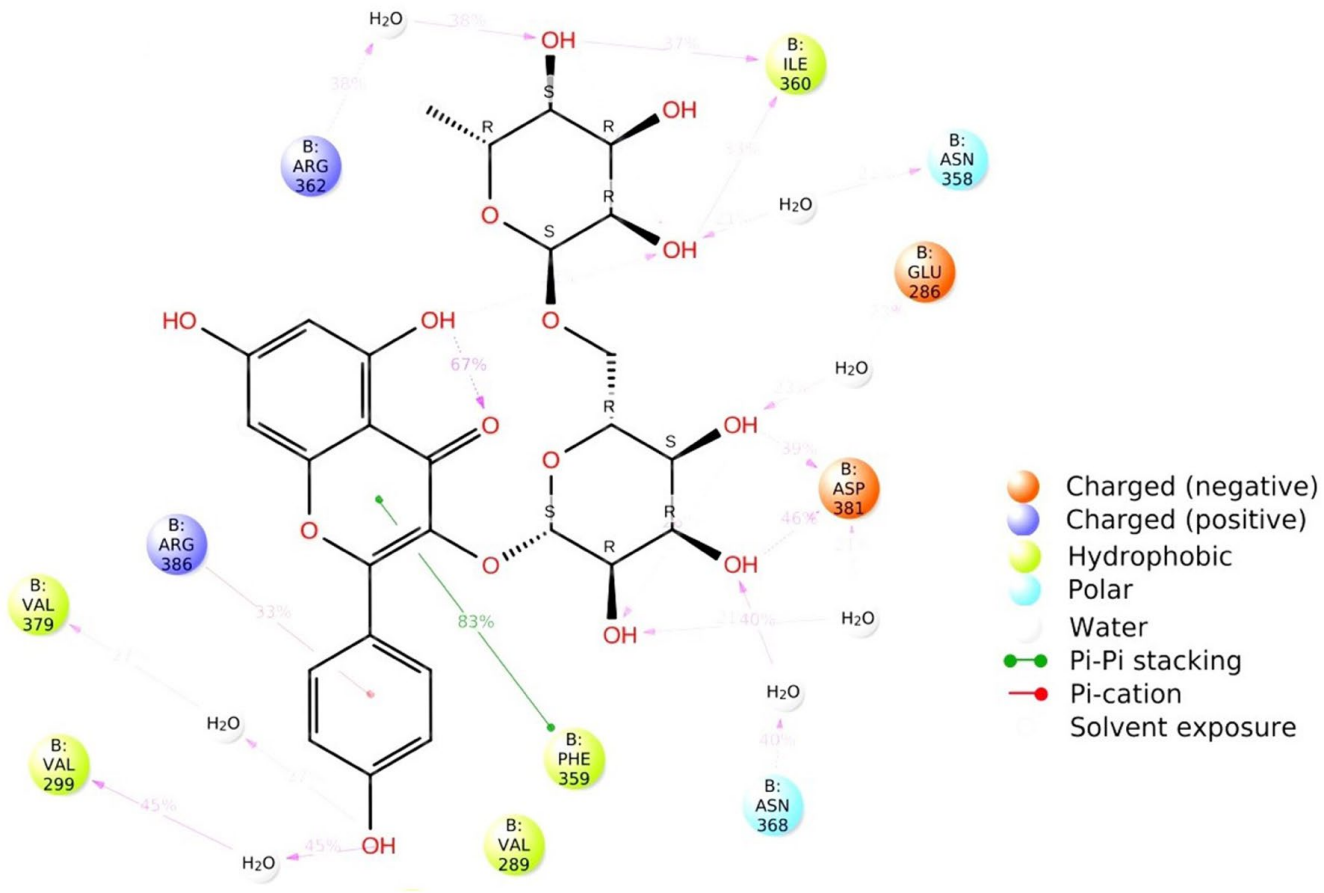
MD simulation analysis of biorobin-1IEP complex 2D interaction diagram.

100 ns MD simulations with the Desmond MD tool were performed to better understand the stability of Phytochemical Biorobin in the binding cavity of C-ABL kinase Domain (PDB ID: 1IEP) (Fig. 7). The root-mean-square deviation (RMSD), root-mean-square fluctuation (RMSF), and protein–ligand interactions were calculated using the MD trajectories.

RMSD serves as a crucial quantitative measure to assess the stability of the protein–ligand complex by quantifying the average displacement of atoms compared to the initial reference frame over a specific period. A lower RMSD value during the MD simulation indicates a more stable protein–ligand complex, while a higher RMSD value suggests lower stability^63,64^.

**Figure 8:**
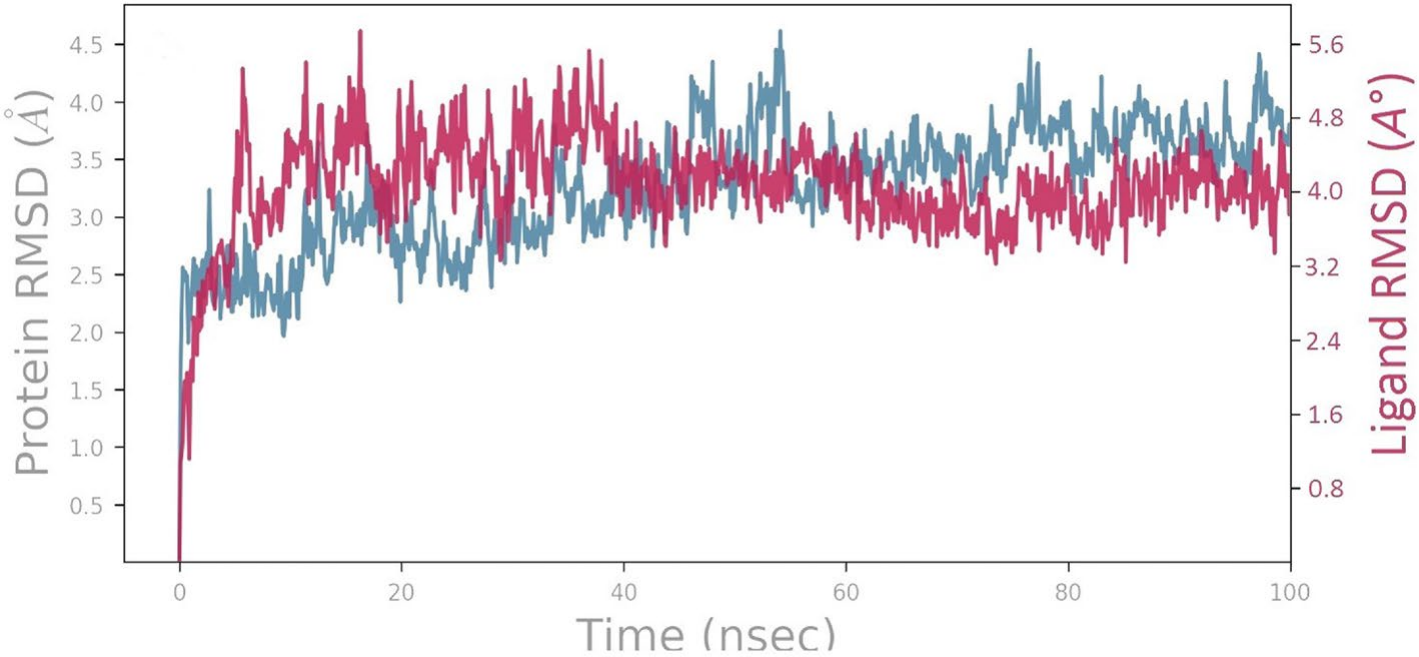
MD simulation analysis of biorobin-1IEP complex RMSD (protein RMSD is shown in blue while RMSD of biorobin is shown in red).

Throughout the simulation, the RMSD of the Cα atoms (shown in blue) of the 1IEP Protein is calculated using the reference frame backbone (Fig. 8). The maximum RMSD value observed for the Cα atoms of the 1IEP Protein was 4.5 Å. Following initial fluctuations, the RMSD remained relatively consistent with minor fluctuations, indicating that the 1IEP-Biorobin complex maintained stability throughout the simulation period. The RMSD plot depicted an increasing trend from the start of the simulation (0–25 ns), followed by minor fluctuations that persisted until the end. Following the initial fluctuation caused by equilibration, the RMSD of the Biorobin in complex with the 1IEP protein system remained within the range of 3.2 Å to 5.6 Å. During the initial 0–49 ns, significant fluctuations in the RMSD were observed, ranging from 3.5 to 5.6 Å. Subsequently, the RMSD exhibited minor fluctuations that were not significant, and the complex remained stable for the remainder of the simulation.

**Figure 9:**
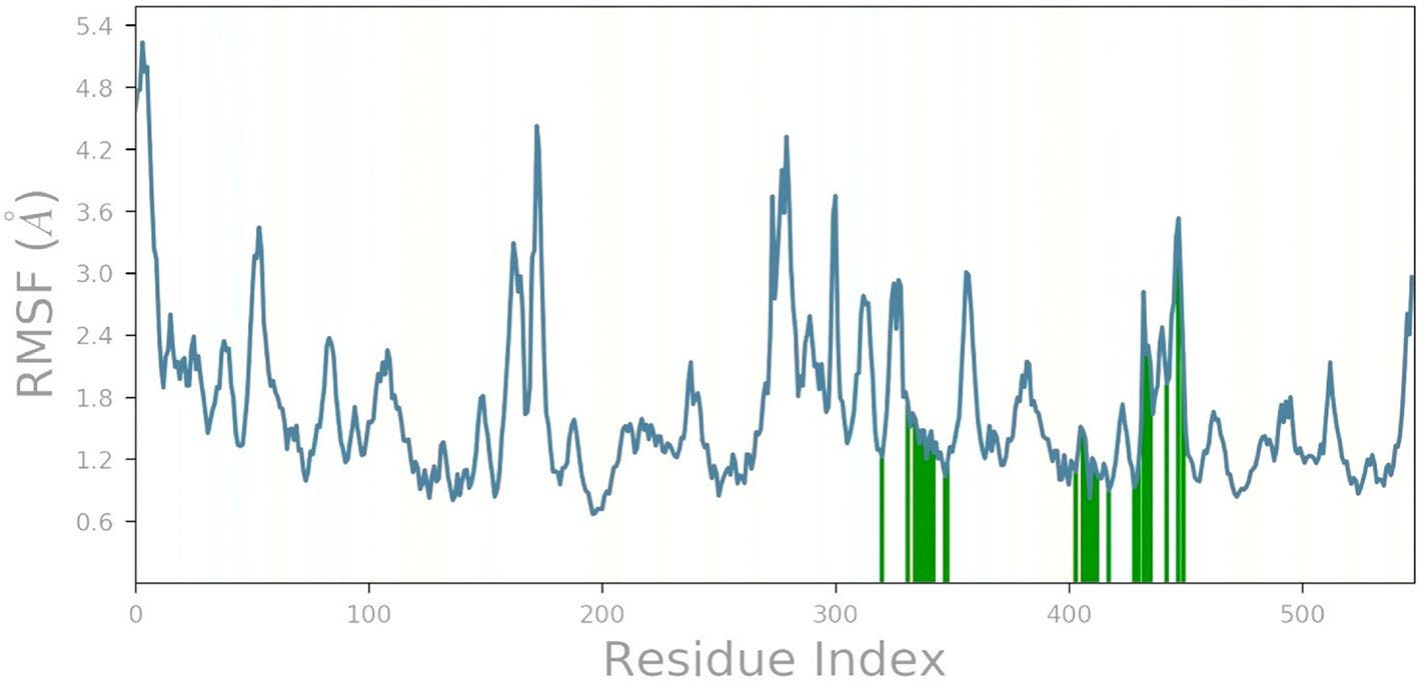
MD simulation analysis of biorobin-1IEP complex Individual amino acids of RMSF.

During simulation, the RMSF parameter helps monitor local conformation changes in the protein chain. Protein RMSF plot analysis provides information about the protein's flexible regions. Compared to other α-helices and β-sheets, which are usually more rigid, both N- and C- terminal regions exhibit higher RMSF values, corresponding to the expected high flexibility of their loop structure^44,65^. Figure 9 shows the RMSF plot of the 1IEP-Biorobin complex. Phytochemical Biorobin was found to interact 28 amino acids of the 1IEP, including Lys271(1.2 Å), Glu282(1.7 Å), Lys285(1.6 Å), Glu286(1.5 Å), AlA288(1.5 Å), VAl289(1.5 Å), MEt290(1.2 Å), Glu292(1.5 Å), Ile293(1.3 Å), Leu298(1.0 Å), VAl299(1.2 Å), Leu354(1.1 Å), Lys357(1.5 Å), Asn358(1.4 Å), Phe359(1.1 Å), Ile360(0.8 Å), His361(1.2 Å), Arg362(1.2 Å), Asp363(1.1 Å), Asn368(0.9 Å), VAl379(0.9 Å), Asp381(1.2 Å), Gly383(2.8 Å), Ser385(2.3 Å), Arg386(2.1 Å), Tyr393(1.9 Å), Gly398(3.5 Å), and Lys400 (2.2 Å). The active site amino acids demonstrated the minor local conformational alteration with lead compound Biorobin (˂ 2.8 Å), except Gly398. This observation validated the binding pocket's structural stability during the simulation.

**Figure 10:**
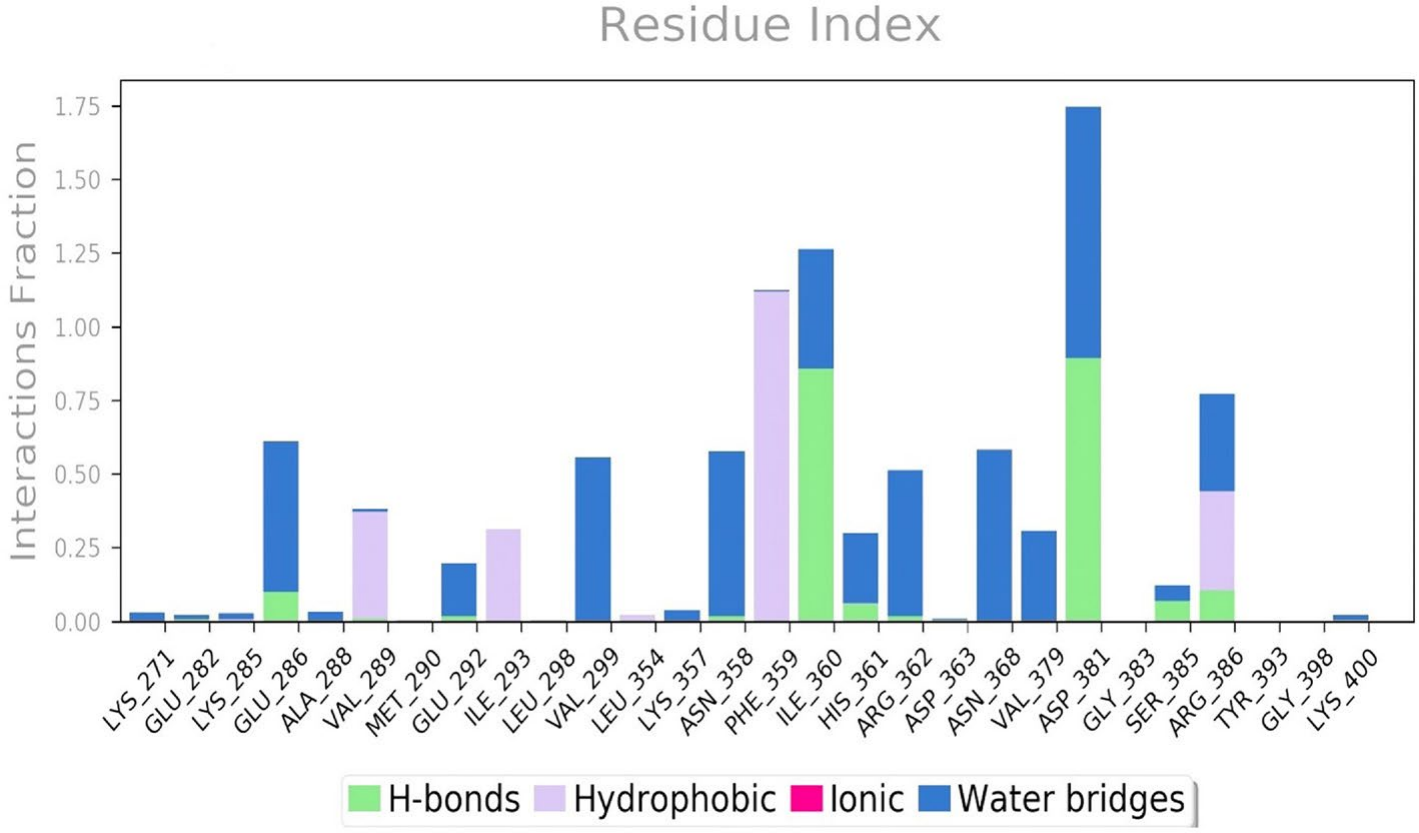
MD simulation analysis of biorobin-1IEP complex protein–ligand contact analysis of MD trajectory.

The analysis of protein–ligand contacts revealed that the residue Phe359 displayed a significant 83% hydrophobic interaction with the chromene scaffold of the lead compound Biorobin. This interaction information is presented in Fig. 10, which shows a histogram of the protein–ligand complex binding interactions throughout the simulation period. Among the amino acids, Ile360 and Asp381 contributed predominantly to the hydrogen bond interactions, accounting for more than 75% of the total interactions. On the other hand, hydrophobic interactions were mainly facilitated by Val289, Ile293, Phe359, and Arg386, contributing to over 20% of the interactions. Interestingly, the interactions observed during the initial docking between the 1IEP Protein and Biorobin were maintained throughout the MD simulation. This consistency suggests that the binding mode prediction achieved during docking was stable and accurately reflected the actual binding interactions in the system.

### Drug likeness and ADMET study

In the clinical assessments, unsuccessful drug discovery and development are primarily due to inadequate bioavailability. The most qualified applicants may be chosen to successfully pass through clinical testing and prevent detention by including crucial ADMET traits early on ^25^. The top five putative phytochemicals were checked for their drug-likeness and ADMET, which has the highest binding affinities in terms of molecular docking (Table 6). These phytochemicals are Biorobin, Irinotecan, Isoorientin 7-glucoside, Armillarin, and Euphornin.

**Table 6:** Preliminary Phytochemical Screening of *Dalbergia sissoo* leaf extracts.

Name of phytochemical	Dock score (kcal/mol)	Drug likeness			ADMET prophecies						
		D	A	L	B	E	R	G	I	AA	S
Biorobin	−9.0	−	2	3	9.13	0.25	0.02	6.28	42.3	−4.59	−
Irinotecan	−8.6	−	2	1	22.16	0.06	0.03	97.63	77.76	−4.21	+
Isoorientin 7-glucoside	−8.4	−	4	3	3.57	0.04	0.02	1.60	39.02	−44.72	_
Armillarin	−7.9	+	2	0	20.54	0.04	0.30	94.29	94.61	−2.47	+
Euphornin	−7.4	−	2	1	30.93	0.04	0.01	97.08	92.29	−1.29	+

( +): qualified; (−): not qualified; *D* drug-likeness, *A* lead likeness violation, *L* Lipinski’s violation, *B* Caco-2, *E* MDCK, *R* BBB, *G* HIA, *I* plasma protein binding, *AA* skin permeability, *S* Pgp inhibition.

Based on the results, it is evident that flavonoid Biorobin exhibited the highest binding affinity against the C-ABL kinase receptor, which is more expressed by the high stability during the interaction with the receptor during extensive 100 ns MD simulation time. This comprehensive in silico study is supported by the positive results in the ADMET study, which suggests that Biorobin has the highest inhibition potential against the malignant growth of the K562 cell line by inhibiting the C-ABL kinase receptor.

### Future perspective

The current study explored the leaf of *Dalbergia sissoo*, a plant used by tribal people to treat cancer, for its potential anticancer phytochemicals. The study aimed to identify the compounds that could be lead molecules for designing and developing novel anticancer drugs by semi-synthetic methods, particularly as an important inhibitor of the C-ABL kinase receptor of cancer cells. This bridging between folk use and modern medicinal systems could lead to the development of potent anticancer agents.

## Conclusion

In vitro antioxidant and anticancer potential of traditional herbal *Dalbergia sissoo* leaves were investigated across six cancer cell lines. The leaf extracts of *Dalbergia sissoo* exhibited their significance as a valuable reservoir of natural antioxidants. Employing an HRLC-MS profiling study, natural bioactive compounds with substantial anticancer effects agaist K562 and A431 cell lines were identified. Within the leaf extracts, phytochemicals such as flavonoids, coumarins, alkaloids, and others demonstrated potent antitumor potential on benign and malignant tumors. Including these bioactive agents in plant extracts further enhances their potential to exhibit chronic diseases like cancer. Thorough in silico investigations like molecular docking, MD simulations, and ADMET, it was determined that Biorobin stands out as the most effective bioactive phytochemical present in *Dalbergia sissoo* leaves for effectively inhibiting the C-ABL kinase receptor in leukemia cell lines. Such comprehensive analyses pave the way for advancing innovative bioactive pharmaceuticals targeting dreaded cancer diseases. These findings can associate the folk tribal use of the leaves of *Dalbergia sissoo* with the future drug design and development of more potent analogs and more optimized formulations of traditional medicines as potent cytotoxic.

### Supplementary Information

Supplementary Information.

## Data Availability

The data that support the findings of this study are available in the supplementary material of this article.
