# Supplementary material for: LC–MS profiling, in vitro and in silico C-ABL kinase inhibitory approach to identify potential anticancer agents from Dalbergia sissoo leaves
Source: Sci Rep. 2024 Jan 2;14:73. doi: 10.1038/s41598-023-49995-1 (PMC10761914; doi:10.1038/s41598-023-49995-1)
Supplement: Supplementary file 1 — Supplementary Information. [file 41598_2023_49995_MOESM1_ESM.docx]

***ELECTRONIC SUPPORTING INFORMATION***

**LC-MS profiling, *in vitro* and *in silico* C-ABL kinase inhibitory approach to identify potential anticancer agents from *Dalbergia sissoo* leaves**

Hem N. Naik^a^, Dilip Kanjariya^a^, Shahnaz Parveen^b^, Iqrar Ahmed^c^, Abha Meena^b^, Harun Patel^d^, Ramavatar Meena^e^ and Smita Jauhari^a,*^

1. Department of Chemistry, SV National Institute of Technology, Surat 395007, Gujarat, India.
2. Molecular Bioprospection Department, CSIR-Central Institute of Medicinal and Aromatic Plants, Lucknow 226015, Uttar Pradesh, India.
3. Department of Pharmaceutical Chemistry, Prof. Ravindra Nikam College of Pharmacy, Gondur, Dhule, 424002, Maharashtra, India.
4. Division of Computer Aided Drug Design, Department of Pharmaceutical Chemistry, R.C. Patel Institute of Pharmaceutical Education and Research, Shirpur, 425405, Maharashtra, India.
5. Natural Product and Green Chemistry Division, CSIR-Central Salt & Marine Chemicals Research Institute, G. B. Marg, Bhavnagar 364002, Gujarat, India.

Corresponding author e-mail address: [sjauhari190@gmail.com](mailto:sjauhari190@gmail.com)

**List of Tables**

**Table. S1** Average Percentage cytotoxicity of plant extracts against six different cell lines

**Table. S2** Reported biological activity of screened phytochemicals from **HN-2** extract

**Table S3**. Minimum inhibitory Concentration of plant extracts agaist different microbial strains

**Table S4.** IC_50_ and percentage inhibition of tested extracts for DPPH radical scavenging property

**List of Figures**

**Figure. S1** Mass spectrum of Maculosin

**Figure. S2** Mass spectrum of Fraxetin

**Figure. S3** Mass spectrum of Isoorientin 7-glucoside

**Figure. S4** Mass spectrum of Biorobin

**Figure. S5** Mass spectrum of Ismine

**Figure. S6** Mass spectrum of Manumycin A

**Figure. S7** Mass spectrum of Celereoin

**Figure. S8** Mass spectrum of Sayanedine

**Figure. S9** Mass spectrum of Armillarin

**Figure. S10** Mass spectrum of Irinotecan

**Figure. S11** Mass spectrum of Euphornin

**Figure. S12** Mass spectrum of Cirsimaritin

**Figure. S13** UV-Vis spectrum of blank (DPPH solution in methanol)

**Figure. S14** Graphical representation of the antimicrobial activity of *Dalbergia sissoo* extracts

**Table. S1** Average Percentage cytotoxicity of plant extracts against six different cell lines

| **Entry** | **% CYTOTOXICITY AVERAGE** | | | | | |
| --- | --- | --- | --- | --- | --- | --- |
|  | **K562** | **PC3** | **A431** | **A549** | **NCIH 460** | **HEK-293T** |
| HN-1 | 28.35±1.72 | 14.31±3.09 | 46.86±8.15 | 14.13±3.61 | 32.18±7.51 | 3.51±10.0 |
| HN-2 | 79.02±4.38 | 9.00±10.07 | 40.71±7.04 | 29.77±8.07 | 48.10±11.8 | 9.06±5.56 |
| HN-3 | 19.84±5.64 | 17.67±3.64 | 8.10±2.17 | 4.80±1.75 | 16.60±7.42 | 9.19±5.22 |
| HN-4 | 50.19±0.45 | 10.84±7.38 | 79.02±4.38 | 13.92±2.74 | 16.36±6.44 | 3.39±6.25 |
| Podophyllotoxin | 59.33±0.69 | -- | -- | -- | -- | -- |
| Doxorubicin | -- | 72.13±4.26 | 86.27±0.28 | 68.43±0.96 | 77.75±2.18 | 71.62±1.01 |

**Table. S2** Reported biological activity of screened phytochemicals from **HN-2** extract

| **Entry** | **Name of phytochemicals** | **Reported activity** | **Reported source** | **References** |
| --- | --- | --- | --- | --- |
| 1 | Maculosin | Antioxidant | *Streptomyces* | 1 |
| 3 | Fraxetin | Anticancer | Source not disclosed | 2 |
| 4 | Isoorientin 7- glucoside | Anticancer | *Eremurus Spectabilis* Leaves | 3 |
| 5 | Biorobin | Anticancer | Source not disclosed | 4 |
| 6 | Ismine | Antitumor | *Hippeastrum*, *Amaryllidaceae* | 5 |
| 7 | Manumycin A | Anticancer | *Streptomyces parvulus* | 6 |
| 8 | Celereoin | Anticancer | *Ammi visnaga* roots | 7 |
| 9 | Sayanedine | Anticancer | *Dalbergia parviflora* | 8 |
| 10 | Armillarin | Cytotoxicity | *Armillaria mellea* | 9 |
| 11 | Irinotecan | Antioxidant, anticancer | *Camptotheca acuminate* | 10 |
| 12 | Euphornin | Antitumor | *Euphorbia helioscopia* | 11 |
| 13 | Cirsimaritin | Anticancer | *Ocimum sanctum, Microtea debilis, Artemisia judaica, Cirsium japonicum,* and *Lithocarpus dealbatus* | 12 |

***In vitro* Antimicrobial Activity**

Four different leaf extracts were examined for antibacterial activity against two bacteria *E. Coli* MTCC 443, and *V. cholerae* MTCC 3906 bacterial strains and for antifungal activity *C. albicans* MTCC 227 using paper disc diffusion technique at Microcare Laboratory, Surat, Gujarat, India ^13^.

**Table S3**. Minimum inhibitory Concentration of plant extracts against different microbial strains

| **Microbial Strains** | **Minimum Inhibitory Concentration (µg/mL)** | | | | | |
| --- | --- | --- | --- | --- | --- | --- |
|  | **HN-1** | **HN-2** | **HN-3** | **HN-4** | **Ampicillin** | **Griseofulvin** |
| *E. coli* | 200 | **100** | 100 | 250 | 100 | - |
| *V. cholerae* | 250 | **100** | 200 | 100 | 100 | - |
| *C. albicans* | 500 | **100** | 200 | 250 | - | 500 |

**Table S4. Free radical scavenging activity of different extracts (**IC_50_ and percentage inhibition)

| **Entry** | **% Inhibition** | **IC_50_ (µg/mL)** | **Entry** | **% Inhibition** | **IC_50_ (µg/mL)** | **Entry** | **% Inhibition** | **IC_50_ (µg/mL)** |
| --- | --- | --- | --- | --- | --- | --- | --- | --- |
| **HN-2** | 9.44±0.47 | **182.21±2.39** | HN-3 | 0.36±0.01 | 1382.45±1.82 | HN-4 | 6.35±0.31 | 248.36±0.81 |
|  | 10.17±0.50 |  |  | 0.63±0.03 |  |  | 6.35±0.31 |  |
|  | 12.62±0.63 |  |  | 1.27±0.06 |  |  | 7.81±0.39 |  |
|  | 14.25±0.71 |  |  | 3.45±0.17 |  |  | 9.71±0.48 |  |
|  | 15.80±0.79 |  |  | 5.08±0.25 |  |  | 10.26±0.74 |  |

**Figure. S1** Mass spectrum of Maculosin


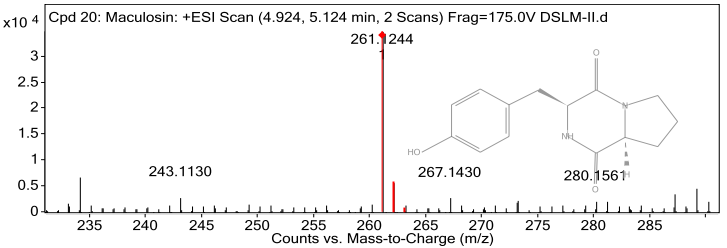


**Figure. S2** Mass spectrum of Fraxetin


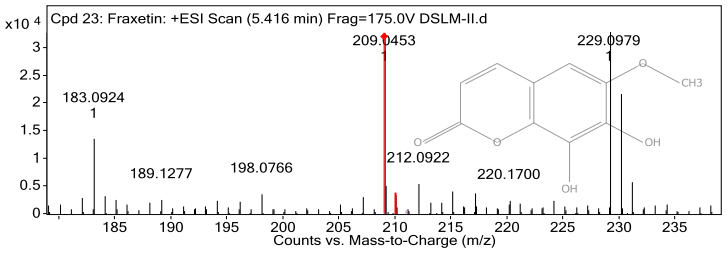


**Figure. S3** Mass spectrum of Isoorientin 7-glucoside


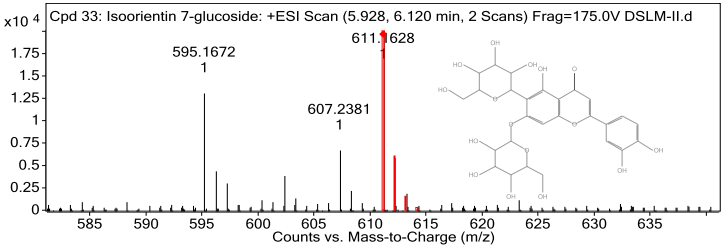


**Figure. S4** Mass spectrum of Biorobin


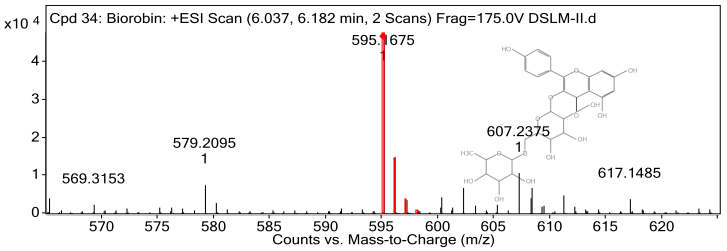


**Figure. S5** Mass spectrum of Ismine


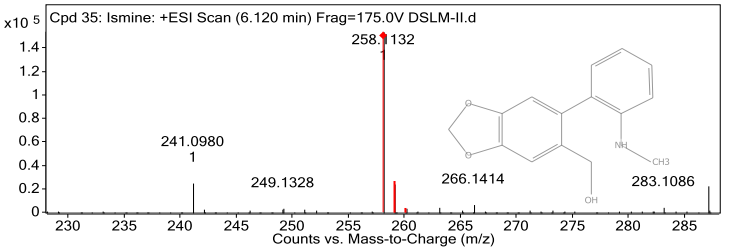


**Figure. S6** Mass spectrum of Manumycin A


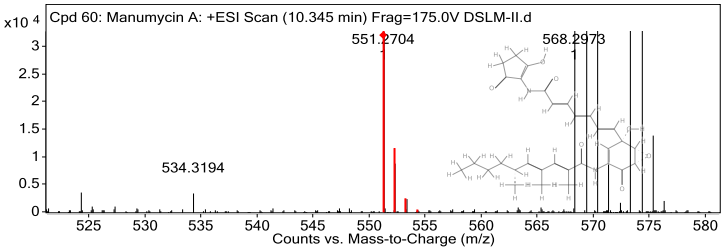


**Figure. S7** Mass spectrum of Celereoin


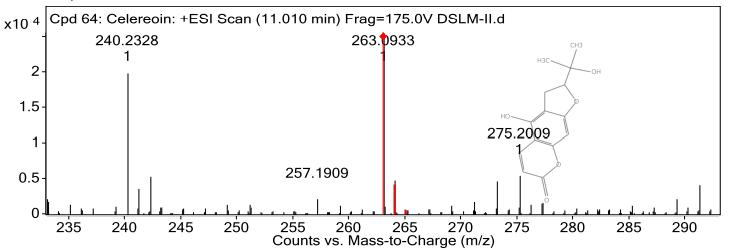


**Figure. S8** Mass spectrum of Sayanedine


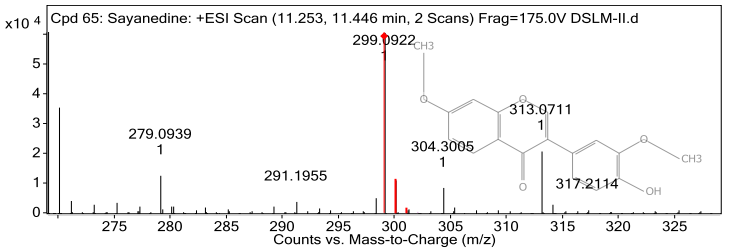


**Figure. S9** Mass spectrum of Armillarin


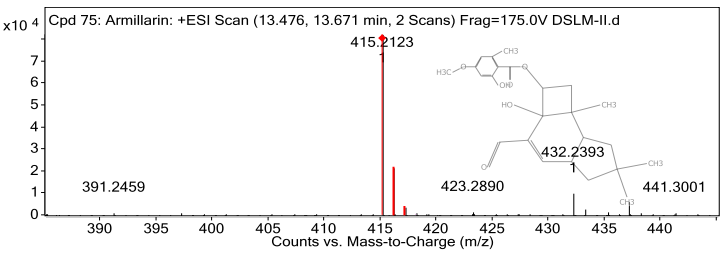


**Figure. S10** Mass spectrum of Irinotecan


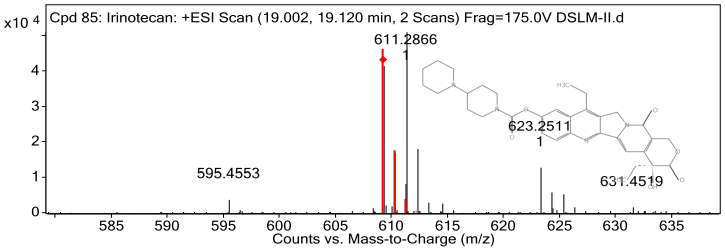


**Figure. S11** Mass spectrum of Euphornin


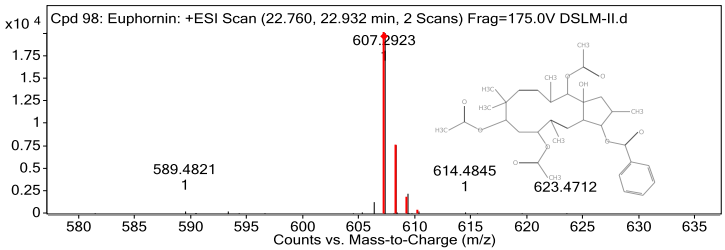


**Figure. S12** Mass spectrum of Cirsimaritin


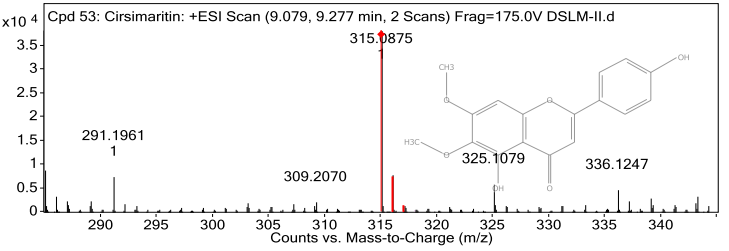


**Figure S13.** UV-Vis spectrum of blank (DPPH solution in methanol)

**Figure S14**. Graphical representation of the antimicrobial activity of *Dalbergia sissoo* extracts

**References**

1. Paudel, B. *et al.* Maculosin, a non-toxic antioxidant compound isolated from Streptomyces sp. KTM18. *Pharm Biol* **59**, 933–936 (2021).

2. Liu, G., Liu, Z., Yan, Y. & Wang, H. Effect of fraxetin on proliferation and apoptosis in breast cancer cells. *Oncol Lett* **14**, 7374–7378 (2017).

3. Gundogdu, G., Dodurga, Y., Elmas, L., Yilmaz Tasci, S. & Karaoglan, E. S. Investigation of the anticancer mechanism of isoorientin isolated from eremurus spectabilis leaves via cell cycle pathways in HT-29 human colorectal adenocarcinoma cells. *Eurasian Journal of Medicine* **50**, 168–172 (2018).

4. Kosmider, B. & Osiecka, R. Flavonoid compounds: A review of anticancer properties and interactions with cis-diamminedichloroplatinum(II). *Drug Dev Res* **63**, 200–211 (2004).

5. Serhan, M. *et al.* Total iron measurement in human serum with a smartphone. *AIChE Annual Meeting, Conference Proceedings* **2019-Novem**, (2019).

6. Tuladhar, A. & Rein, K. S. Manumycin A Is a Potent Inhibitor of Mammalian Thioredoxin Reductase-1 (TrxR-1). *ACS Med Chem Lett* **9**, 318–322 (2018).

7. Ahmed, S. S. T. *et al.* Metabolomics of the secondary metabolites of Ammi visnaga L. roots (family Apiaceae) and evaluation of their biological potential. *South African Journal of Botany* (2022) doi:10.1016/j.sajb.2022.01.011.

8. Umehara, K. *et al.* Estrogenic constituents of the heartwood of Dalbergia parviflora. *Phytochemistry* **69**, 546–552 (2008).

9. Li, Z. *et al.* Structure, cytotoxic activity and mechanism of protoilludane sesquiterpene aryl esters from the mycelium of Armillaria mellea. *J Ethnopharmacol* **184**, 119–127 (2016).

10. Fujita, K. I., Kubota, Y., Ishida, H. & Sasaki, Y. Irinotecan, a key chemotherapeutic drug for metastatic colorectal cancer. *World J Gastroenterol* **21**, 12234–12248 (2015).

11. Li, X. Q., Bai, Y. L., Zhang, D. L., Jiao, H. S. & He, R. X. Euphornin reduces proliferation of human cervical adenocarcinoma hela cells through induction of apoptosis and G2/M cell cycle arrest. *Onco Targets Ther* **11**, 4395–4405 (2018).

12. Pathak, G. *et al.* Cirsimaritin, a lung squamous carcinoma cells (NCIH-520) proliferation inhibitor. *J Biomol Struct Dyn* **39**, 3312–3323 (2021).

13. Zala, A. R., Rajani, D. P. & Kumari, P. Design, synthesis, molecular docking and antimicrobial and antimycobacterial activities of novel hybrid of coumarin-cinnamic acids. *Chemical Data Collections* **39**, 100862 (2022).
